# Artificial Intelligence perception and its influence on the Psychological Distress of Healthcare Professional

**DOI:** 10.12669/pjms.42.1.12787

**Published:** 2026-01

**Authors:** Badr Alnasser, Rakesh Kumar

**Affiliations:** 1 Badr Alnasser, Ph.D., Department of Health Management, College of Public Health and Health Informatics, University of Ha’il, Ha’il, Saudi Arabia; 2 Rakesh Kumar, Ph.D., Department of Health Management, College of Public Health and Health Informatics, University of Ha’il, Ha’il, Saudi Arabia

**Keywords:** AI perception, Psychological Distress, Technology Readiness, Healthcare professional, Saudi Arabia

## Abstract

**Objectives:**

Th research examines how AI perception (AIP) affect psychological distress of healthcare professional in Kingdom of Saudi Arabia (KSA). The study also investigates the moderating role of technology readiness (TRD).

**Methodology:**

This research adopts cross-sectional design. A self-administrative, close ended, survey was used to collect primary data. Total 411 healthcare professionals voluntarily participated from public hospitals of the Hail Health Cluster, in KSA. The survey was carried out through convenience sampling method. Data was processed through SPSS 27 version. Analysis consisted of demographic summary, descriptive analysis and regression analysis through Hayes process.

**Results:**

A total of 411 healthcare professionals participated, with the majority being male (60.8%) and aged 36-45 (41.4%). Additionally, the descriptive statistics revealed adequate reliability for all variables including AIP, DASS-21 and TRD. Moreover, the regression analysis showed that AIP significantly influenced DASS-21 (*β* = -0.2194, *p* = 0.0099). Finally, the TRD strengthen the negative impact of AIP on DASS-21 (*β* = 0.1547, *p* = 0.0000). The model’s R-Square values indicated a strong fit, with significant improvements in prediction.

**Conclusion:**

The study highlights that AI perception significantly impacts psychological distress, particularly Depression and Anxiety, among healthcare professionals. While technology readiness significantly strengthens this relationship. Future research should explore other potential moderating factors, such as organizational support, to further understand the impact of AI perception in healthcare settings.

## INTRODUCTION

The integration of AI promises the optimization of healthcare practices and their systems.^1^ However, there is a lack of understanding on how healthcare professional perceives the AI. Conceptually speaking, AI perception may be referred to as the user’s attitude for the limitations and advantages for acknowledging AI practices in their operations.^2^ The perception of AI is an important driver for technology adoption in healthcare.^3^ AI shape how users engage with new technology.^4^ Therefore, understand the AI perception is necessary. Furthermore, it is always demanded from healthcare sector to provide better care to their patients with improved efficiency. In these ways, AI is highlighted as a most promising tool to enhance the operational efficiency of any organization.^5^ However, a positive attitude of healthcare staff and their readiness towards new technology adoption is necessary for the successful implementation of AI in their system.

Psychological distress which comprised off stress, anxiety, and depression among healthcare staff has become a growing issue. Increasing level of distress can have a negative effect on patient’s care along with workforce productivity.^6^ Additionally, the increasing level of stress is also related to higher level of turnover intentions of staff in healthcare service providing.^7^ Therefore, it is more important to understand how readiness of adopting a new technology can impact the relationship between AI perception and distress. The readiness of technology may be referred to as the willingness of professionals for accepting the implementations of new technology in their regular operations.^8^ Higher level of readiness increases to adopt AI based technology. This trend may help to reduce the stress and increases job related satisfaction among professionals.^9^ Therefore, this research aimed to investigate the role of AI perception, and technology readiness in determining the distress. Furthermore, technology readiness would also help to examine the moderating impact for AI perception on distress. The findings of the study offer novel insights for healthcare industry of KSA.

## METHODOLOGY

The study considered a cross-sectional research design to investigate the research questions of how AI perception affects distress in healthcare sector of KSA. It further investigates the moderating role of technology readiness for the above impact. The respondents for data collection of this study includes the professionals from public healthcare settings in KSA. These include the working people from, and various departments (such as; Allied Health service, Nursing, Medicine, Administration, and others). A voluntary participation was confirmed from 411 respondents. As a result, the data was collected during February-March 2025. The participants were included in this study based on their voluntary responses as per their willingness.

### Ethical Consideration:

An Ethical Review Committee (RTC) from University of Ha’il (UOH) approved this study using No. H-2024-351; Dated: May 27, 2024. The participants were informed the aim of this study and the procedures. Confidentiality was observed during the study. Participants provided their informed consent after being fully briefed about the study. The survey formally started after the respondents agreed to take part voluntarily. In this way, the data was collected for this study.

### Informed consent:

Prior to their inclusion in the study, informed consent was obtained from all participants. Participants were informed that they could withdraw from the study at any time without facing any repercussions.

### Inclusion and Exclusion Criteria:

The study considered healthcare professional from KSA as a primary inclusion criterion. Additionally, those who did not wish to participate were excluded from the study.

### Instrument and Variables:

A closed-ended, self-administered survey was the major data collection instrument, which comprised four sections. Section one captured demographic characteristics including gender, age, education, field of work and work exposure. Section two, which assessed AIP, comprised 11 items that were intended to capture perception regarding AI application in the field of healthcare, for instance, “I believe that the application of AI in my field of specialization could facilitate enhanced delivery of patient care.” This variable serves as the independent variable in this study and was adapted from Kumar, Singh.^10^ The third section focused on Psychological Distress included depression, anxiety and stress (seven items each) serving as the dependent variable in this study. To measure psychological distress, DASS-21 scale was adapted from Henry, Crawford.^11^ The fourth section assessed Technology Readiness using 12 items like, “New technologies contribute to a better quality of life,”. It was adapted from Parasuraman and Colby.^12^ It evaluates the moderator’s impact on the relationship between AI perception and psychological distress.

### Sampling Method and Data Gathering:

A convenience sampling method was involved to survey healthcare professionals from the hospitals that were chosen from the Hail health cluster. Estimates of the minimum sample size were computed based on a 90% comprehension of AI for healthcare, a 5% error margin and a 95% confidence interval. The required minimum sample size was 410 participants but distributed to 450 respondents for robustness, of whom 411 respondents completed the survey. It represents 91% response rate. A survey link was sent to participants via email, allowing them to complete the questionnaire at their convenience. Participants were briefed on the study’s objectives and their responses were treated with confidentiality.

### Data analysis:

The data analysis includes respondent’s summary of their personal attributes (e.g., age, discipline, and years of experience). Additionally, the data analysis further includes the descriptive statistics of variables of the study (e.g., mean, standard deviations, and Cronbach alpha). Finally, regression analysis was conducted utilizing Hayes’ Process for the identification of the direct association of workforce performance with AI perception. Even, the technology-readiness based moderating impact was also computed through the analysis.

## RESULTS

A total of 411 participants completed the survey on demographic characteristics and their responses regarding AIP, TRD and DASS-21. Among these, 60.8% were male (n = 250) and 39.2% were female (n = 161). The majority of respondents were aged 36-45 years (41.4%, n = 170), followed by 26-35 years (26.3%, n = 108). Those aged 46-55 years and 18-25 years represented 18.0% (n = 74) and 8.8% (n = 36), respectively, and 5.6% (n = 23) were older than 55 years. Regarding job discipline, 39.9% worked in medicine (n = 164), 23.8% in nursing (n = 98) and 11.4% in allied health (n = 47). In terms of work experience, 26.5% had 9-10 years (n = 109) and 25.3% had more than 10 years (n = 104). Table-I summarizes the demographic characteristics of the respondents.

**Table-I:** Respondent’s Summery.

Characteristics	Categories	N	%
Gender	Male	250	60.80%
	Female	161	39.20%
Age	18-25	36	08.80%
	26-35	108	26.30%
	36-45	170	41.40%
	46-55	74	18.00%
	> 55	23	05.60%
Job Discipline	Medicine	164	39.90%
	Nursing	98	23.80%
	Allied health	47	11.40%
	Administration	86	20.90%
	Others	16	03.90%
Work Experience	0-2	36	08.80%
	3-4	24	05.80%
	5-6	47	11.40%
	7-8	91	22.10%
	9-10	109	26.50%
	more than 10	104	25.30%

The descriptive statistics for the study variables showed that AI perception had a mean score of 3.52, with a reliability coefficient (Cronbach’s alpha) of 0.787 and a range between 1.00 and 5.00. The standard deviation for AIP was 1.32. Technology readiness had a mean score of 3.69 and a reliability coefficient of 0.728, with a range between 1.00 and 5.00 and a standard deviation of 1.15. Psychological distress (DASS-21) had a mean score of 2.62, with a reliability coefficient of 0.842, a range between 1.00 and 4.00 and a standard deviation of 0.99. All three scales demonstrated acceptable internal consistency, with DASS-21 showing the highest reliability coefficient.

For examining the aim of this study, a regression analysis was performed using Hayes’ PROCESS. It was conducted to examine the influence of AI perception and technology readiness on psychological distress (comprising depression, anxiety and stress). AIP had a significant negative effect on depression (coefficient = -0.2797, p = 0.0016), anxiety (coefficient = -0.3213, p = 0.0005) and overall psychological distress (DASS-21; coefficient = -0.2194, p = 0.0099). However, AIP had a non-significant effect on stress (coefficient = -0.0572, p = 0.5398). TRD had a negative but non-significant effect on depression (coefficient = -0.1378, p = 0.0796) and anxiety (coefficient = -0.1496, p = 0.0681), and also a non-significant effect on stress (coefficient = -0.0130, p = 0.8760) and DASS-21 (coefficient = -0.1001, p = 0.1856). The interaction term (AIP × TRD) significantly predicted all dimensions of DASS-21, with positive coefficients for depression (coefficient = 0.1693, p < 0.001), anxiety (coefficient = 0.1813, p < 0.001) and stress (coefficient = 0.1135, p < 0.001). Finally, the inclusion of interaction-term (moderating variable) has significantly improved the model’s overall fit. It is evident through the value of coefficient of determination (or simply *R^2^*).

**Table-II:** Descriptive Statistics and Reliability.

Variables	Statements	Reliability	Observations	Min	Max	Mean	Std_Dev
AIP	11	0.78	411	1.00	5.00	3.52	1.31
TRD	12	0.72	411	1.00	5.00	3.68	1.15
DASS-21	21	0.84	411	1.00	4.00	2.62	0.99

Note: AIP = AI Perception, TRD = Technology Readiness.

**Table-III:** Regression Analysis (using process by Hayes).

Effects	Depression		Anxiety		Stress		DASS-21	
	Coefficient	P-value	Coefficient	P-value	Coefficient	P-value	Coefficient	P-value
AIP	-0.27	0.001	-0.32	0.001	-0.05	0.539	-0.21	0.009
TRD	-0.13	0.079	-0.14	0.068	-0.01	0.876	-0.10	0.185
AIP× TRD	0.16	0.000	0.18	0.000	0.11	0.000	0.15	0.000
Constant	1.77	0.000	1.75	0.000	1.29	0.000	1.61	0.000
F	215.31	0.000	212.66	0.000	177.79	0.000	231.48	0.000
R^2^	0.61	0.000	0.61	0.000	0.56	0.000	0.63	0.000
R^2^Δ	0.04	0.000	0.05	0.000	0.02	0.000	0.04	0.000

***Note:*** AIP = AI Perception, TRD = Technology Readiness.

## DISCUSSION

This research provides novel empirical evidence for the impact of AI perception, and technology readiness on psychological distress for healthcare setting in KSA. The findings indicated that AI perception strongly affected psychological distress (including depression, and anxiety). This finding matches with similar results of studies indicating a negative impact of AI for distress.^13^,^14^ The negative impact of AI perception for distress advocates the positive view of AI for decreasing the mental health for healthcare staff.^15^,^16^ However, the findings indicated that AI perception does not strongly impact the stress as a component of distress. This inconsistency might have arisen due to nature of stress among healthcare professional in KSA context. This type of stress may be derived by complex demands of patients, shortages of staff, constraints of tie, and workload issues. These issues may be controlled using specific staff attitudes rather than technology adoption like AI. There are a number of factors that may play important role for controlling stress rather than AI. These include role ambiguity, emotional labor, and workload.^17^-^19^ Therefore, it is obvious that immediate environmental and organizational demands may significantly remain constant regardless of favorable AI perception among healthcare professionals. This interpretation is consistent with previous literature emphasizing the dominant role of structural and contextual stressors in shaping stress outcomes among healthcare professionals. 17-19

TRD had a negative but non-significant effect on Depression and Anxiety. This finding is consistent with studies suggesting that technology readiness alone does not directly reduce psychological distress.^20^,^21^ Healthcare professionals may feel psychologically and behaviorally prepared to engage with AI, but such readiness does not automatically translate into lower distress unless AI tools are actually implemented in a supportive way, integrated into workflows and perceived as genuinely helpful for reducing workload and enhancing efficiency. The lack of significant effects on stress and psychological distress further supports this idea. It suggests that readiness to adopt AI does not necessarily correlate with reduced stress in healthcare professionals.^22^

The interaction between AIP and TRD significantly predicted all components of psychological distress (DASS-21). In particular, the moderation results indicate that technology readiness strengthens the negative relationship between AI perception and psychological distress, such that healthcare professionals who both perceive AI positively and feel ready to use it report lower levels of distress. It infers that AI perception, as well as technology readiness both affect psychological distress significantly. It aligns with a matching result from study with inferred that readiness of technology strongly moderates the effect of AI on wellbeing.^23^ It emphasized on how both variables are important in decreasing the distress. This finding suggests that customized training, organizational support, and continued technical support in the form of technical readiness can enhance the impact of AI. Therefore, it can facilitate how we can use AI effectively in healthcare and clinical practices.

This research made a significant contribution by filling a research gap for an underexamined context of healthcare sector of KSA.^8^,^24^-^26^ Further research can consider emotional labor, workload, and organizational culture as contextual factors to study additional insight. These factors can examine how AI perception, and readiness of technology may affect the distress in different settings.^27^,^28^ Furthermore, future studies could also consider turnover intentions, job satisfaction, and burnout as additional outcomes to execute some valuable research. Finally, the future studies can consider cross-regional context for the healthcare studies to increase the generalizability.

### Strengths:

This research study has a number of strengths in terms of addressing specific context, multidimensional constructs, and inclusion of a moderator. Firstly, this study addresses an under-investigated context of healthcare professional in KSA. It provides specific empirical evidence for this context for the impact of AI perception, and technology readiness on psychological distress. This evidence is related to non-western context and are specifically related to setting of middle eastern region. Secondly, the construct like DASS-21 is a multidimensional measure of psychological distress which is used in this study. This measure consists of three dimensions; depression, anxiety, and stress. Finally, another strength of this study is the use of technology readiness as the moderator. The existing literature primarily focused on the direct impacts of factors related to AI. However, this research explicitly considered a moderator in the form of technology readiness. It indicates how and when AI perception may be more advantageous.

### Limitations:

This research acknowledges a number of limitations that need to be addressed. The first limitation is design of study (e.g., cross-sectional). This design avoids causal inferences for the impact of AI perception, and technology readiness on psychological distress. Therefore, a longitudinal research design is needed. This research design validates the directions of these impacts. Furthermore, the data used for the analysis of this study were obtained through the self-reported measures of participants. It may cause the issue of comment method bias as well as social desirability for the role of AI perception for mental health. Finally, the findings of the study may also limit its generalizability due to its relevance towards the healthcare sector of KSA.

## CONCLUSION

The research concludes with the negative impact of AI perception on psychological distress of healthcare professionals in KSA. However, the findings of this research could not conclude a significant impact of AI perception on stress. The results suggest to consider emotional labor and workload as the additional contextual factors for predicting stress level. Moreover, the readiness of technology didn’t impact the distress. It concludes that readiness of technology along may not emphasize in mitigating the burden on mental health. However, it strengthens the impact of AI perception in reducing psychological distress. Future research should investigate organizational and cultural moderators. This will help better understand the impact of AI perception and readiness on healthcare professionals’ psychological well-being in diverse healthcare settings.
